# Disrupted Control-Related Functional Brain Networks in Drug-Naive Children with Attention-Deficit/Hyperactivity Disorder

**DOI:** 10.3389/fpsyt.2017.00246

**Published:** 2017-11-21

**Authors:** Jiejie Tao, Xueyan Jiang, Xin Wang, Huiru Liu, Andan Qian, Chuang Yang, Hong Chen, Jiance Li, Qiong Ye, Jinhui Wang, Meihao Wang

**Affiliations:** ^1^Department of Radiology, First Affiliated Hospital of Wenzhou Medical University, Wenzhou Medical University, Wenzhou, China; ^2^Center for Cognition and Brain Disorders, Hangzhou Normal University, Hangzhou, China; ^3^Zhejiang Key Laboratory for Research in Assessment of Cognitive Impairments, Hangzhou, China; ^4^Department of Radiology, Yancheng First Peoples’ Hospital, Yancheng, China; ^5^Department of Psychiatry, First Affiliated Hospital of Wenzhou Medical University, Wenzhou Medical University, Wenzhou, China

**Keywords:** brain network, executive control, graph theory, long-range connectivity, resting-state fMRI

## Abstract

Attention-deficit/hyperactivity disorder (ADHD) is a common neurodevelopmental disease featuring executive control deficits as a prominent neuropsychological trait. Executive functions are implicated in multiple sub-networks of the brain; however, few studies examine these sub-networks as a whole in ADHD. By combining resting-state functional MRI and graph-based approaches, we systematically investigated functional connectivity patterns among four control-related networks, including the frontoparietal network (FPN), cingulo-opercular network, cerebellar network, and default mode network (DMN), in 46 drug-naive children with ADHD and 31 age-, gender-, and intelligence quotient-matched healthy controls (HCs). Compared to the HCs, the ADHD children showed significantly decreased functional connectivity that primarily involved the DMN and FPN regions and cross-network long-range connections. Further graph-based network analysis revealed that the ADHD children had fewer connections, lower network efficiency, and more functional modules compared with the HCs. The ADHD-related alterations in functional connectivity but not topological organization were correlated with clinical symptoms of the ADHD children and differentiated the patients from the HCs with a good performance. Taken together, our findings suggest a less-integrated functional brain network in children with ADHD due to selective disruption of key long-range connections, with important implications for understanding the neural substrates of ADHD, particularly executive dysfunction.

## Introduction

Attention-deficit/hyperactivity disorder (ADHD) is one of the most common neurodevelopmental disorders in children and adolescents, with a prevalence of approximately 5.3% worldwide (1, 2). The core behavioral symptoms of ADHD are inappropriate patterns of inattention and/or hyperactivity/impulsivity that seriously affect individual learning and social advantages (3), thus imposing a heavy strain on the affected individuals and society.

Considerable neurophysiological research documents that ADHD is associated with disturbed executive function, a series of higher-order, top-down cognitive control processes that allow flexible, goal-directed behavior (4), such as motor inhibition, vigilant attention, set switching, planning, and working memory (5–10). Evidence from neuroimaging studies further shows that regions implicated in these processes, such as the dorsolateral prefrontal cortex, dorsal anterior cingulate cortex, and parietal regions, are abnormal in ADHD patients (11–15). These findings collectively suggest executive control dysfunction as a key characteristic of ADHD.

Recently, a dual-network model of human task control was proposed (16–18). One network is the frontoparietal network (FPN), which mainly includes the dorsolateral prefrontal cortex, intraparietal sulcus, inferior parietal lobule, and mid-cingulate, and supports adaptive control in initiation and adjustment. The other network is the cingulo-opercular network (CON), which mainly consists of the dorsal anterior cingulate, anterior insula/frontal operculum, anterior prefrontal cortex, and thalamus, and supports stable set-maintenance functions. In addition to these two networks, several cerebellar regions showing error-related activity across tasks (16) are functionally connected to the FPN and CON (17, 19), suggesting possible feedback of error signals from the cerebellar network (CN). Another network is the default mode network (DMN), which is mainly composed of the bilateral posterior cingulate, inferior parietal cortex, and ventromedial prefrontal cortex. The DMN routinely shows decreased activity during goal-directed tasks (20) and exhibits an anti-correlation with the FPN in spontaneous neural fluctuations in the absence of external stimuli (21). Moreover, accumulating evidence demonstrates disrupted interactions between the DMN and FPN/CON in ADHD (22–25).

Here, we investigated functional connectivity patterns among these four control-related networks in 46 drug-naive ADHD children and 31 age-, gender-, and intelligence quotient (IQ)-matched healthy controls (HCs) by combining resting-state functional MRI (R-fMRI) and graph-based approaches. Specifically, 34 regions of interest comprising the four networks were defined in this work according to a previously published study (26). Given the local-to-distributed functional architecture among these networks during development (26), we hypothesized that children with ADHD would exhibit a less-integrated organization due to possible developmental delays caused by the disease.

## Materials and Methods

### Participants

A total of 69 children with ADHD (6–13 years old) and 44 HCs (6–13 years old) participated in the study. All participants were right-handed according to the Annett Hand Preference Questionnaire (27) and were Han Chinese. The ADHD children were recruited from the First Hospital Affiliated to Wenzhou Medical University of Mental Health Center, and the healthy children were recruited from a local primary school during the period from March 2012 to November 2014. None of the ADHD patients received any medication before data collection. After a complete explanation of the study protocol, all children agreed to take part in this study, and written informed consent was obtained from their guardians. This study was approved by the local ethical committee of the First Hospital Affiliated to Wenzhou Medical University.

Diagnosis of ADHD was determined independently by two experienced clinical psychiatrists (CY and HC, who have more than 15 years of experience in clinical psychiatry) according to the Structured Clinical Interview for Diagnostic and Statistical Manual of Mental Disorders, Fourth Edition criteria (DSM-IV) and a semi-structured diagnostic interview, the Schedule for Affective Disorders and Schizophrenia for School-Age Children-Present and Lifetime Version. The parents of the children in the ADHD group scored their children using Conner’s Parent Symptom Questionnaire—Chinese revised version (28). This scale includes the hyperactivity/impulsivity symptoms of ADHD and comorbidities. Specifically, a total of 48 items are included that are related to six aspects: (1) conduct problems; (2) learning problems; (3) physical and psychological problems; (4) impulsivity–hyperactivity score; (5) anxiety; and (6) hyperactivity index score. The questionnaire uses a 4-point scale ranging from 0 to 3: “never” is rated as 0, “occasionally” as 1, “often” as 2, and “always” as 3. The exclusion criteria included (1) left-handedness; (2) a history of stimulants or any other drugs or therapy for the disorder; (3) a full-scale IQ score less than 80 according to the Wechsler Intelligence Scale for Chinese Children-Revised (29); (4) a history of head trauma with loss of consciousness; (5) a history of neurological disorders or other severe diseases, such as pediatric stroke and seizure disorder; (6) a history of psychiatric disorders including affective disorders, emotional disorders, oppositional defiant disorder, Tourette’s syndrome, conduct disorder, or any other Axis I psychiatric disorder. The healthy children were also screened by the same psychiatrists according to the Structured Clinical Interview for DSM-IV with the same exclusion criteria as the ADHD children.

Thirty-six children were excluded due to excessive head motion during the functional MRI data acquisition (34 children, see below for details) or IQ scores < 80 (2 children). Therefore, 46 patients (6–13 years old, 38 males) and 31 controls (6–12 years old, 22 males) were included in the final analysis. Table 1 summarizes demographic and clinical information of these participants.

**Table 1:** Demographic and clinical characteristics of all participants.

	ADHD (*n* = 46)	HCs (*n* = 31)	*P* value
Age (years)	8.48 ± 1.89 (6–13)	8.84 ± 1.57 (6–12)	0.247^a^
Gender (M/F)	38/8	22/9	0.269^b^
IQ	119 (82–145)	120 (95–141)	0.531^c^
Hyperactivity index score	1.45 (0.4–2.8)	0.40 (0–1.5)	<0.001^c^
Impulsive score	1.54 ± 0.63 (0.5–3)	0.50 ± 0.48 (0–1.75)	<0.001^a^

*Values are presented as mean ± standard (minimum − maximum) or median (minimum − maximum) depending on the normality*.

*ADHD, attention-deficit/hyperactivity disorder; HCs, healthy controls; M, male; F, female; IQ, intelligence quotient*.

*^a^P-values were obtained using two-sample t-tests*.

*^b^P-value was obtained using a chi-square test*.

*^c^P-values were obtained using Wilcoxon rank sum tests*.

### Image Acquisition

All MRI scans were performed on a GE signal HDx 3 T MR scanner with an eight-channel phased-array head coil (GE Medical Systems, Milwaukee, WI, USA). During the entire scanning procedure, all subjects were in a supine position with their heads snugly fixed by foam pads to reduce head movement. Whole-brain R-fMRI data depicting blood oxygen level-dependent (BOLD) contrast were obtained using a gradient-echo echo-planar imaging sequence with the following parameters: 31 axial slices, slice thickness = 4 mm, slice gap = 0.2 mm, repetition time (TR) = 2,000 ms, echo time (TE) = 30 ms, flip angle = 90°, matrix size = 64 × 64, and field of view (FOV) = 192 mm × 192 mm. The R-fMRI lasted 8 min in total, and 240 volumes were obtained for each participant. During the R-fMRI scans, the participants were instructed to keep their eyes closed, relax their minds, and remain as still as possible without falling asleep. It was confirmed that none of the participants fell asleep during the scan by subjective report. Individual structural images were also acquired using a 3D T1-weighted SPGR sequence with the following parameters: 176 sagittal slices, slice thickness = 1.0 mm, no gap, TR = 2,530 ms, TE = 3.39 ms, inversion time = 1,100 ms, FA = 7°, matrix size = 256 × 256, FOV = 256 mm × 256 mm.

### Data Preprocessing

Resting-state functional MRI data preprocessing was performed using the GRETNA toolbox (30) and SPM12.^1^ After removal of the first 10 volumes to allow T1 equilibration effects, individual R-fMRI data were corrected for within-volume time acquisition differences between slices (Sinc interpolation) and inter-volume head motion (six-parameter rigid transform). Thirty-four children (21 patients and 13 HCs) were excluded from further analysis due to excess head motion (>3 mm translation or >3° rotation in any direction). We further examined several summary measures of both gross (the maximum and root mean square) and subtle (mean frame-wise displacement) head motion profiles for the remaining participants and found no significant between-group differences (all *P*s > 0.241). The corrected images were then spatially normalized into standard Montreal Neurological Institute (MNI) space via segmentation and resampled to 3-mm cubic voxels. Because pediatric brains differ significantly from adult brains, we used the CCHMC Pediatric Brain Templates^2^ during the normalization to avoid the introduction of systematic biases (31). The normalized images were subsequently subjected to removal of linear trends and temporal band-pass filtering (0.01–0.08 Hz). Finally, several nuisance signals including the white matter signal, cerebrospinal fluid signal, global signal, and 24 head motion parameters (32) were regressed out from each voxel’s time course.

### Functional Connectivity Matrix

**Figure 1:**
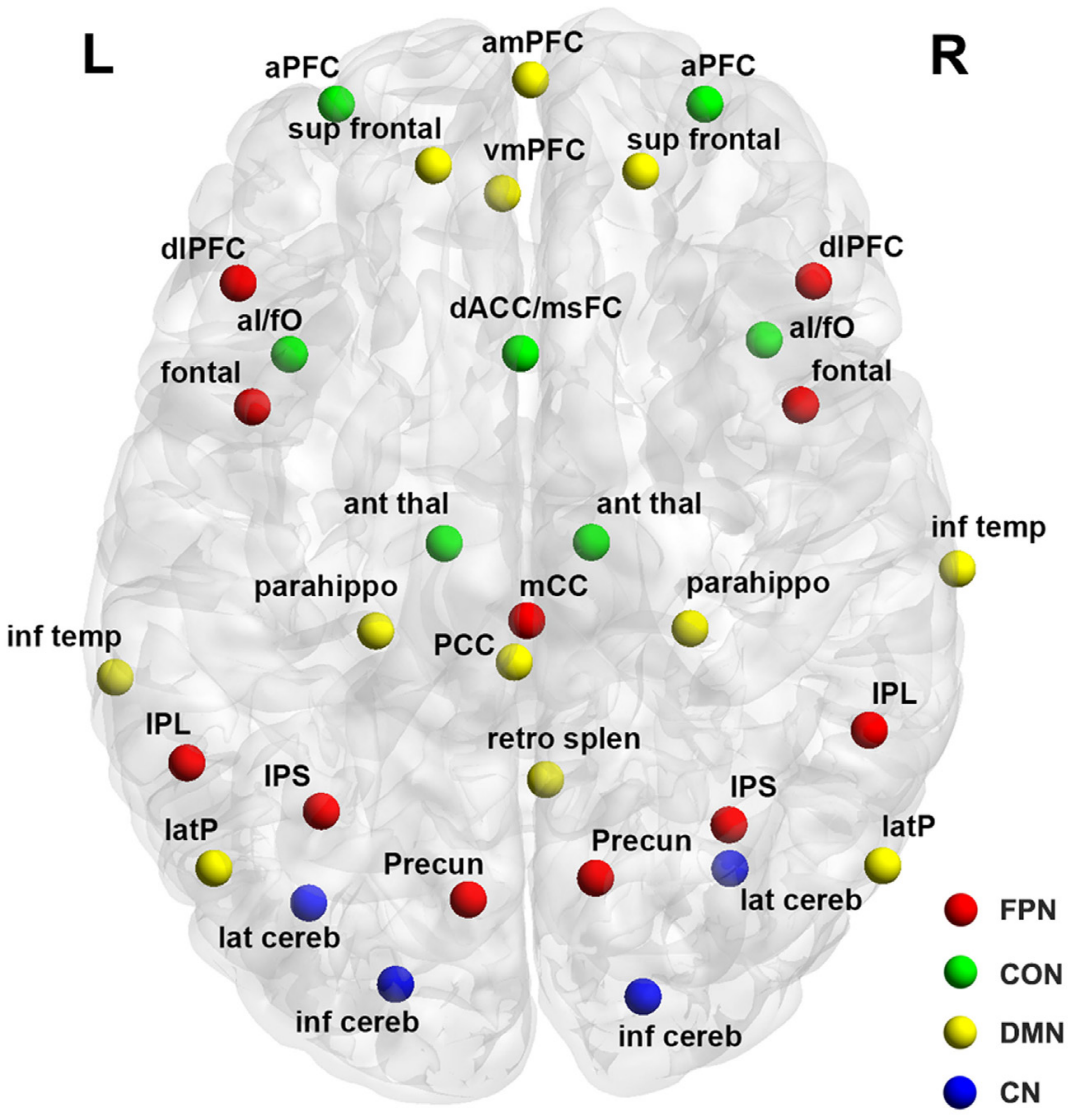
Surface representation of the anatomical locations of regions included in the frontoparietal network (FPN), cingulo-opercular network (CON), cerebellar network (CN), and default mode network (DMN). See Table 3 for regional abbreviations.

A total of 34 previously published regions comprising the four functional networks (i.e., FPN, CON, CN, and DMN) were used in this study (Figure 1; Table 2). Specifically, we first plotted 34 spheres (radius = 6 mm) in the brain in the standard MNI space, which were centered at the coordinates reported in Ref. (26). We then extracted the average BOLD time series within each sphere and calculated pairwise Pearson correlation coefficients among the resulting 34 average time series, thus generating a 34 × 34 correlation matrix for each participant. Given the ambiguous interpretation (33–35), detrimental effects on test–retest reliability (36, 37) and distinct connectivity patterns (38), negative correlations were excluded (set to 0) from the correlation matrices in all subsequent analyses. Indeed, all connectivity and topological analyses listed below revealed no significant between-group differences for negative correlation matrices (i.e., positive correlations were set to 0).

**Table 2:** Regions of interest.

Name	Abbreviation	Montreal Neurological Institute Coor	Category	Color
Dorsolateral prefrontal cortex	dlPFC	[−45 28 31]	FPN	R
Dorsolateral prefrontal cortex	dlPFC	[48 28 30]	FPN	R
Frontal	frontal	[−43 8 36]	FPN	R
Frontal	frontal	[46 8 34]	FPN	R
Mid-cingulate cortex	mCC	[2 −26 32]	FPN	R
Inferior parietal lobule	IPL	[−53 −49 42]	FPN	R
Inferior parietal lobule	IPL	[57 −44 46]	FPN	R
Intraparietal sulcus	IPS	[−32 −57 49]	FPN	R
Intraparietal sulcus	IPS	[34 −59 44]	FPN	R
Precuneus	Precun	[−8 −71 44]	FPN	R
Precuneus	Precun	[13 −68 46]	FPN	R
Anterior medial prefrontal cortex	amPFC	[2 61 13]	DMN	Y
Ventromedial prefrontal cortex	vmPFC	[−2 43 −11]	DMN	Y
Superior frontal cortex	sup frontal	[−15 47 49]	DMN	Y
Superior frontal cortex	sup frontal	[20 46 49]	DMN	Y
Inferior tempora	inf templ	[−65 −35 −17]	DMN	Y
Inferior tempora	inf templ	[71 −18 −21]	DMN	Y
Parahippocampal	parahippo	[−23 −28 −19]	DMN	Y
Parahippocampal	parahippo	[28 −28 −18]	DMN	Y
Posterior cingulate cortex	pCC	[0 −33 40]	DMN	Y
Lateral parietal	latP	[−49 −66 43]	DMN	Y
Lateral parietal	latP	[59 −66 41]	DMN	Y
Retro splenia	retro splen	[5 −52 9]	DMN	Y
Lateral cerebellum	lat cereb	[−34 −72 −29]	CN	B
Lateral cerebellum	lat cereb	[34 −66 −31]	CN	B
Inferior cerebellum	inf cereb	[−20 −85 −33]	CN	B
Inferior cerebellum	inf cereb	[20 −87 −33]	CN	B
Anterior prefrontal cortex	aPFC	[−29 57 7]	CON	G
Anterior prefrontal cortex	aPFC	[30 57 15]	CON	G
Anterior insula/frontal operculum	aI/fO	[−37 17 0]	CON	G
Anterior insula/frontal operculum	aI/fO	[40 19 −3]	CON	G
Anterior thalamus	ant thal	[−12 −14 5]	CON	G
Anterior thalamus	ant thal	[12 −14 6]	CON	G
Dorsal anterior cingulate/medial superior frontal cortex	dACC/msFC	[1 17 45]	CON	G

*DMN, default mode network; FPN, frontoparietal network; CON, cingulo-opercular network; CN, cerebellar network; R, red; Y, yellow; B, blue; G, green*.

### Topological Analysis

#### Threshold Selection

In the framework of graph theory, the correlation matrices derived above can be viewed as weighted graphs or networks comprising nodes and edges, with nodes representing the spheres and edges representing inter-sphere connectivity (correlation coefficients as weights). Prior to the topological characterization of these networks, a thresholding procedure was used to convert them into binary networks, in which the inter-nodal connectivity weights were either 0 or 1, indicating the absence or presence of an edge between two nodes, respectively. In the current study, two thresholding methods were employed: a correlation thresholding approach and a sparsity thresholding approach. For the correlation thresholding method, the same correlation threshold was applied to all individual correlation matrices such that connectivity weights greater than the threshold were set to 1, and others were set to 0. This method generates networks with different numbers of edges across participants due to possible differences in their overall connectivity strength and thus allows an examination of the absolute network organization. By contrast, for the sparsity thresholding approach, a subject-specific correlation threshold was used to ensure the same number of edges (i.e., the same network sparsity or density, defined as the ratio of the number of actual edges divided by the maximum possible number of edges in a network) across participants. This thresholding method therefore allows an examination of the relative network organization by imposing on each network the same number of edges or wiring cost for compensatory adaptations. These two thresholding strategies are complementary and together provide a comprehensive method to test the network organization (39). Therefore, network measurements derived using these two different thresholding approaches quantify distinct aspects of topological network organization and may reveal inconsistent findings in diseases (39, 40). Specifically, the threshold values were determined according to the following criteria: (1) the average degree (see below for the definition of nodal degree) over all nodes of each thresholded network was larger than 2 × log(*N*), with *N* denoting the number of nodes (i.e., 34) (41); and (2) all thresholded networks had no isolated nodes. This procedure generated a maximum correlation threshold of 0.12 and a minimum sparsity threshold of 0.34 across the participants. All further topological analyses were thus based on networks thresholded with these two threshold values. We also examined the effects of different threshold values on our findings (correlation threshold = 0.08, 0.10, 0.14, and 0.16; sparsity threshold = 0.30, 0.32, 0.36, and 0.38), and largely comparable results were observed (data not shown).

#### Global Network Measures

We calculated the global efficiency, local efficiency and modularity to characterize the overall global topology of the derived brain networks. Mathematically, the global efficiency of a network *G* with *N* nodes is defined as follows (42):

$$E_{\text{glob}}(G)=\frac{1}{N(N-1)}\sum_{i\neq j\in G}\frac{1}{d_{ij}}$$

where *d_ij_* is the shortest path length between node *i* and node *j* and is calculated as the smallest number of edges among all possible paths from node *i* to node *j*. The global efficiency measures the ability of parallel information transfer over the entire network. The local efficiency of *G* is calculated as follows (42):

$$E_{\text{loc}}(G)=\frac{1}{N}\sum_{i\in G}E_{\text{glob}}(G_{i})$$

where *E*_glob_(*G_i_*) is the global efficiency of *G_i_*, the subgraph composed of the neighbors of node *i* (i.e., nodes linked directly to node *i*). The local efficiency reflects how well the network exchanges information locally or how much the network is fault tolerant. The modularity, *Q*, for a given partition, *p*, of *G* is defined as:

$$Q(p)=\sum_{s=1}^{N_{M}}\left[\frac{l_{s}}{L}-{\left(\frac{d_{s}}{2L}\right)}^{2}\right]$$

where *N_M_* is the number of modules, *L* is the total number of connections in *G, l_s_* is the number of connections between nodes in module *s*, and *d_s_* is the sum of the nodal degree (see below for the definition of nodal degree) for nodes in module *s*. Modularity quantifies the difference between the number of intra-module links of the actual network and that of a random network in which connections are linked at random (43). The aim of the module identification process is to identify a specific partition, *p*, that yields the largest network modularity, *Q*_max_. Here, we detected the modular structure using a spectral optimization algorithm (44) implemented using the Brain Connectivity Toolbox.^3^ The number of modules was also recorded.

To determine whether the brain networks exhibited significantly non-random organization, for each participant, the global network measures (local efficiency, global efficiency, and modularity) were normalized by dividing them by the corresponding measures derived from 100 random networks. The random networks were generated using a topological rewiring algorithm that preserved the same number of nodes, edges, and degree distributions as the real brain networks (45, 46). A network showing larger local efficiency and approximately equal global efficiency or larger modularity than random networks is said to be small-world or modular, respectively (41).

#### Nodal Network Measures

For each node, we calculated the nodal degree, nodal efficiency, and nodal betweenness to capture their roles in the brain network. Specifically, for a given node *i* in network *G*, the nodal degree is defined as the number of links connected to it:

$$k_{i}=\sum_{j\in G}a_{ij}$$

where *a_ij_* is 1 or 0 and indicate the presence or absence of an edge between node *i* and node *j*. The nodal efficiency is defined as the average shortest path length between node *i* and all other nodes in the network (47):

$$e_{i}=\frac{1}{N-1}\sum_{j\neq i\in G}\frac{1}{d_{ij}}$$

where *d_ij_* is the shortest path length between node *i* and node *j* in *G*. The nodal betweenness is defined as the number of shortest paths between pairs of other nodes that pass through node *i* (48):

$$b_{i}=\sum_{m\neq i\neq n\in G}\frac{\sigma_{mn}(i)}{\sigma_{mn}}$$

where σ*_mn_* is the total number of shortest paths from node *m* to node *n*, and σ*_mn_*(*i*) is the number of shortest paths from node *m* to node *n* that pass through node *i*.

The uses and interpretations of these global and nodal network measures can be found in Ref. (49).

### Statistical Analysis

#### Between-Group Differences in Demographic and Clinical Variables

Age, IQ, hyperactivity index score and impulsive score were analyzed with two-sample *t*-tests or Wilcoxon rank sum tests depending on whether the data were normally distributed (Lilliefors test). Gender distribution was examined with a chi-square test.

#### Between-Group Differences in Functional Connectivity

To localize the interregional connectivity that differed between the ADHD children and HCs, we utilized a network-based statistic (NBS) approach (50). Briefly, a *t*-statistic matrix was first derived by an edge-wise between-group comparison (two-sample *t*-test) of the functional connectivity strength (i.e., correlation coefficient) for positive connections that existed in at least 50% of the participants. A primary threshold (*P* < 0.05) was then applied to the *t*-statistic matrix to identify supra-threshold connections, within which all connected components and their sizes (i.e., the number of edges included in these components) were identified and determined. To estimate the significance of each component, a null distribution of the maximal connected component size was empirically derived using a permutation approach (10,000 permutations). For each permutation, all subjects were reallocated randomly into two groups, and two-sample *t*-tests were conducted for the same set of connections mentioned above. The same primary threshold (i.e., *P* < 0.05) was then used to generate supra-threshold connections, within which the maximal connected component size was recorded. After these permutations, an empirical distribution of the maximal connected component size was obtained. Finally, for a connected component of size M found in the right grouping of controls and patients, the corrected *P* value was determined by calculating the proportion of the 10,000 permutations for which the maximal connected component was larger than M. The effects of age, gender, IQ, and head motion (maximum, root mean square, and mean frame-wise displacement) were controlled during the NBS analysis.

We further examined whether the ADHD-related functional connectivity alterations were dependent on anatomical distance. First, we first calculated the proportion of long-range connections that were included in the identified NBS component. For a given connection, the anatomical distance was calculated as the Euclidean distance between regional centroids of two regions linked by the connection, and connections with an anatomical distance >75 mm were regarded as long-range connections (51, 52). Then, we compared the mean anatomical distance of all connections included in the NBS component with that of the other connections within the entire network (two-sample *t*-test). Finally, given the imbalance in the number of connections showing or not showing ADHD-related alterations (30 versus 531, see Results), we further performed the following simulation-based statistical analysis. First, we randomly selected 30 connections from the entire network and calculated their mean anatomical distance. The connections were chosen to ensure that they linked the same number of nodes as the observed real NBS component and formed a connected component. This procedure was implemented 10,000 times to generate an empirical null distribution of the mean anatomical distance. The *P* value for the real observation (i.e., the mean anatomical distance of observed real NBS component) was computed as the proportion of the 10,000 times for which the mean anatomical distance was larger than the real observation.

#### Between-Group Differences in Network Topological Measures

For brain network measures (global and nodal), a nonparametric permutation test was used. In brief, for each metric, we initially calculated the between-group differences in their mean values. An empirical distribution of the differences was then obtained by randomly reallocating all values to two groups and recomputing the mean differences between the two randomized groups (10,000 permutations). The 95th percentile points of the empirical distribution were used as critical values in a one-tailed test to determine whether the observed real-group differences occurred by chance. Age, gender, IQ, and head motion (maximum, root mean square, and mean frame-wise displacement) were also controlled.

#### Brain–Clinical Relationships

Rank partial correlations were used to examine the relationships between the network metrics that showed significant between-group differences and clinical variables (hyperactivity index score and impulsive score) in the ADHD patients and HCs separately. Age, gender, IQ, and head motion (maximum, root mean square, and mean frame-wise displacement) were treated as confounding covariates.

### Classification Analysis

Using the receiver operating characteristic (ROC) curve, we performed a preliminary analysis to determine whether the identified network alterations might serve as biomarkers for diagnosing ADHD. This analysis was performed using public MATLAB codes.^4^

## Results

### Demographic and Clinical Characteristics

As shown in Table 1, there were no significant differences in age (*P* = 0.247), gender (*P* = 0.269), or IQ (*P* = 0.531) between the two groups. The ADHD patients showed higher hyperactivity index (*P* < 0.001) and impulsive scores (*P* < 0.001).

### Disrupted Functional Connectivity in ADHD Patients

**Figure 2:**
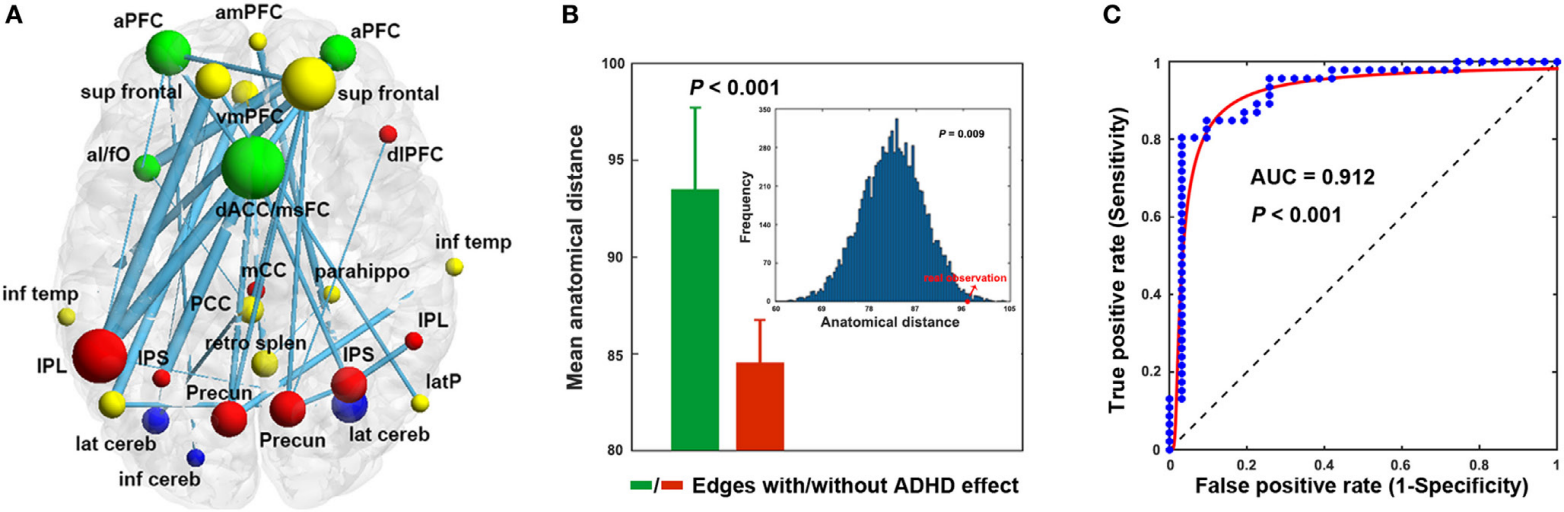
Decreased functional connectivity **(A)**, anatomical distance **(B)**, and classification **(C)**. The nodal colors and regional abbreviations in **(A)** were the same as those in Figure 1. Nodal sizes are proportionate to nodal degrees within the component [i.e., the number of edges showing attention-deficit/hyperactivity disorder (ADHD)-related alterations] and edge widths are proportionate to the extent of between-group differences (i.e., absolute *t* values) in **(A)**. AUC, area under the curve. See Table 3 for regional abbreviations.

Using the nonparametric NBS approach, a single connected component comprising 30 connections linking 26 nodes was identified that showed decreased functional connectivity in the ADHD children compared with the HCs (*P* = 0.018, corrected) (Figure 2A; Table 3). This component was predominantly involved in the inferior parietal lobule, precuneus, intraparietal sulcus, superior frontal cortex, anterior prefrontal cortex, dorsal anterior cingulate/medial superior frontal cortex, and lateral cerebellum, most of which are components of the DMN (11/26, 42.3%) and FPN (8/26, 26.9%). Interestingly, the altered connectivity in the ADHD patients was mainly inter-network (18/30, 60%), long-range (23/30, 76.7%) connections. Further statistical analyses showed that the altered connections were associated with significantly longer anatomical Euclidean distances (two-sample *t* test, *P* < 0.001; simulation-based statistical analysis, *P* = 0.009; Figure 2B) than connections that did not exhibit ADHD effects.

**Table 3:** Decreased functional connectivity in the ADHD children compared to healthy controls.

Region A	Region B	*P*-value	Distance (mm)	Category
IPL.L	dACC/msFC	0.002	85.386	FPN-CON
IPL.L	sup frontal.L	0.002	104.643	FPN-DMN
IPS.L	dACC/msFC	0.003	80.696	FPN-CON
aPFC.R	aI/fO.L	0.003	79.736	CON-CON
vmPFC	latP.L	0.004	130.281	DMN-DMN
sup frontal.R	inf templ.L	0.008	134.846	DMN-DMN
amPFC	lat cereb.R	0.010	138.559	DMN-CN
aPFC.R	dACC/msFC	0.011	58.500	CON-CON
Precun.L	inf templ.R	0.012	115.336	FPN-DMN
vmPFC	retro splen	0.013	97.096	DMN-DMN
Precun.L	dACC/msFC	0.015	88.591	FPN-CON
IPL.R	IPS.R	0.015	27.383	FPN-FPN
aPFC.L	sup frontal.R	0.019	65.390	CON-DMN
IPS.R	dACC/msFC	0.019	83.252	FPN-CON
IPS.R	Precun.R	0.020	23.245	FPN-FPN
sup frontal.R	pCC	0.020	82.324	DMN-DMN
Precun.L	sup frontal.R	0.020	120.936	FPN-DMN
aPFC.L	dACC/msFC	0.021	62.998	CON-CON
Precun.R	sup frontal.R	0.022	114.397	FPN-DMN
latP.L	lat cereb.R	0.022	111.618	DMN-CN
sup frontal.L	latP.R	0.023	134.610	DMN-DMN
mCC	lat cereb.L	0.026	83.918	FPN-CN
aPFC.L	inf cereb.L	0.027	147.951	CON-CN
IPL.L	aPFC.L	0.037	114.410	FPN-CO
dlPFC.R	Precun.R	0.038	103.820	FPN-FPN
IPL.L	lat cereb.R	0.044	115.178	FPN-CN
aI/fO.L	pCC	0.044	73.509	CO-DMN
IPL.L	aPFC.R	0.044	137.908	FPN-CON
parahippo.R	retro splen	0.047	43.527	DMN-DMN
sup frontal.L	lat cereb.L	0.048	144.127	DMN-CN

*L, left; R, right; DMN, default mode network; FPN, frontoparietal network; CON, cingulo-opercular network; CN, cerebellar network*.

*For regional abbreviations, refer to Table 2*.

### Alterations of Absolute Network Organization in ADHD Patients

**Figure 3:**
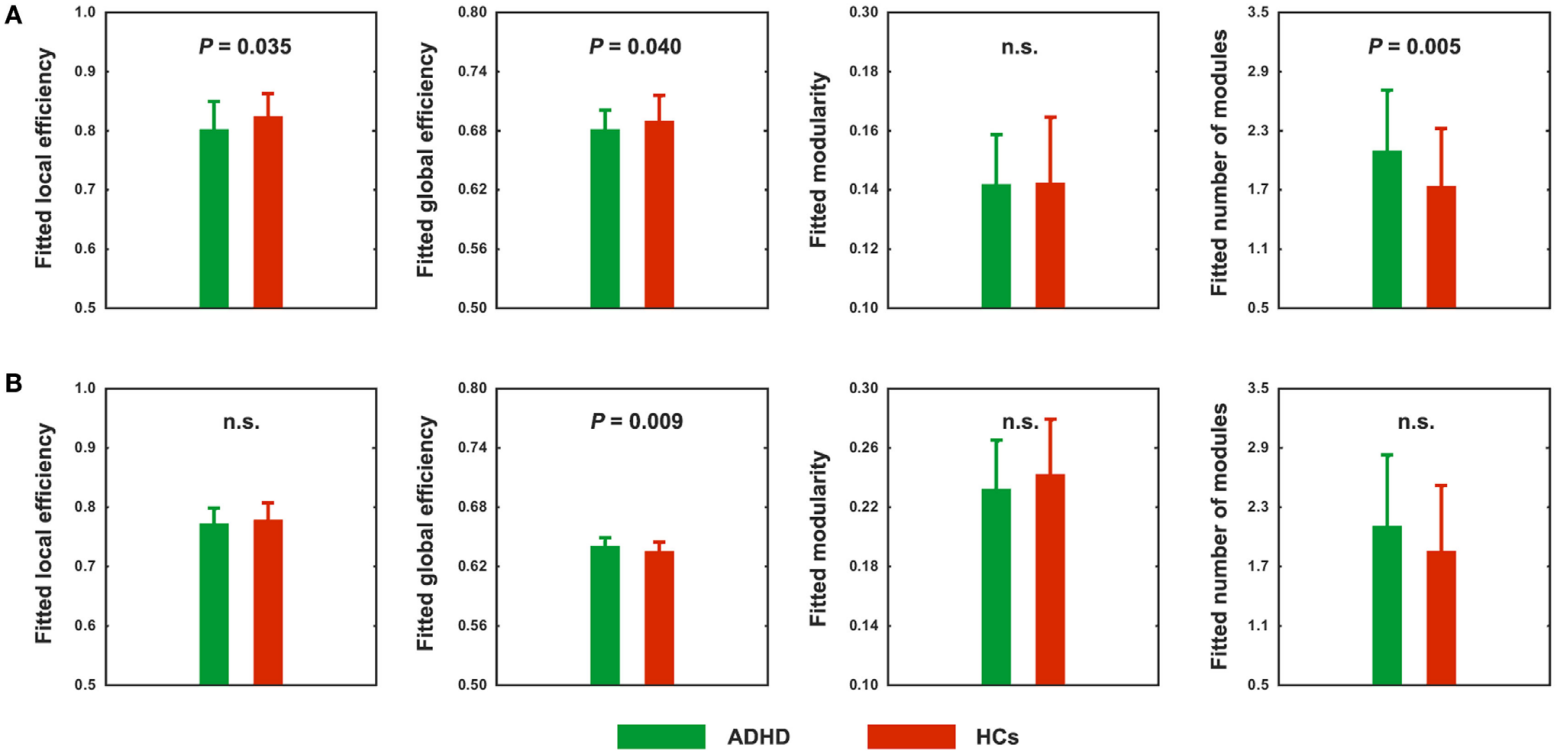
Between-group differences in absolute **(A)** and relative **(B)** network topology. n.s., non-significant.

Using the correlation thresholding approach, significantly fewer connections were found in the networks of ADHD children than in the HCs (network density = 0.337 ± 0.044 for the HCs and 0.317 ± 0.037 for the ADHD children; *P* = 0.020). Topological analyses revealed economical small-world and modular organization in both the ADHD (normalized local efficiency = 1.235 ± 0.115; normalized global efficiency = 0.977 ± 0.009; normalized modularity = 1.544 ± 0.211) and HC (normalized local efficiency = 1.200 ± 0.106; normalized global efficiency = 0.976 ± 0.014; normalized modularity = 1.649 ± 0.258) groups. However, between-group comparisons showed that the local and global efficiency were significantly decreased in the ADHD children compared to the HCs (*P* = 0.035 and 0.040, respectively) (Figure 3A). In addition, the ADHD children had more functional modules in their brain networks than the HCs (*P* = 0.005).

### Alterations of Relative Network Organization in ADHD Patients

Using the correlation thresholding approach, we found that the absolute network organization was disrupted in children with ADHD. However, this method resulted in different network densities (i.e., number of connections) between the ADHD children and HCs, which may confound the between-group comparisons (53, 54). Thus, we further examined relative network organization by using a sparsity thresholding approach, which ensured the same network density across participants. With this method, economical small-world and modular organizations were again observed for the ADHD and HC groups (normalized local efficiency = 1.167 ± 0.057 and 1.171 ± 0.073, normalized global efficiency = 0.983 ± 0.010 and 0.976 ± 0.014, and normalized modularity = 1.553 ± 0.210 and 1.647 ± 0.248; respectively). However, after controlling for between-group differences in the number of connections, the ADHD children exhibited significantly increased global efficiency (*P* = 0.009) (Figure 3B). No significant differences were observed in any other global measures.

### Nodal Characteristics of Functional Brain Networks

No significant differences were found at any node for any nodal metric of the degree, efficiency, or betweenness between the ADHD and HC groups (*P* > 0.05, false discovery rate corrected).

### Brain–Clinical Relationship

**Figure 4:**
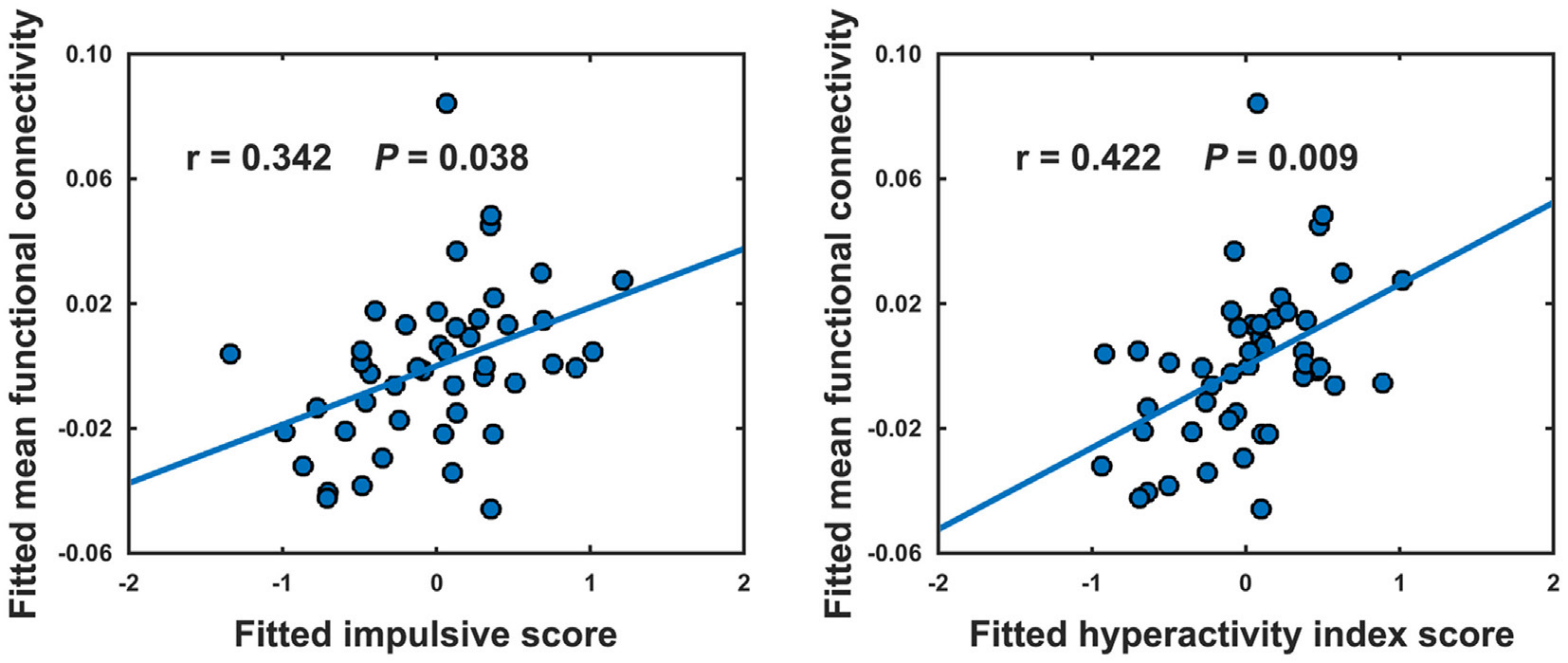
Relationships between the mean strength of decreased functional connectivity in attention-deficit/hyperactivity disorder patients and clinical variables. The relationships were estimated using rank partial correlations with age, gender, and intelligence quotient as covariates.

No significant correlations were observed in the HCs (*P* > 0.05). In the ADHD children, only the mean functional connectivity strength within the NBS-based connected component exhibited significantly positive correlations with the impulsive score (*r* = 0.342, *P* = 0.038) and hyperactivity index score (*r* = 0.422, *P* = 0.009) (Figure 4).

### Network-Based Differentiation of ADHD Children from the HCs

Among the network measures that showed ADHD-related alterations, the mean functional connectivity strength within the NBS-based connected component exhibited the highest power (area under the curve = 0.912, *P* < 10^−3^), with a sensitivity of 84.8% and a specificity of 90.3% (cutoff value = 0.200) for distinguishing the ADHD patients from the HCs (Figure 2C). As such, 39 of the 46 patients with ADHD and 28 of the 31 HCs were classified correctly.

## Discussion

By combining R-fMRI and graph-based approaches, this study investigated the topological organization of four control-related networks in drug-naive children with ADHD. Significantly decreased functional connectivity was observed in the ADHD children that (1) predominantly involved the DMN and FPN; (2) mainly involved across-network long-range connections; (3) correlated with the patients’ clinical symptoms; and (4) exhibited excellent power for disease classification. Topologically, a more segregated organization with decreased network efficiency and increased number of modules was observed in the ADHD patients. Overall, these findings indicate disrupted network organization in ADHD, which has important implications for understanding executive dysfunction in the disease.

Widespread functional connectivity decreases were observed in the ADHD children that primarily involved the DMN and FPN. Fair and colleagues explored the interregional functional connectivity among 12 DMN regions and found that these regions were less integrated in ADHD children than HCs (55). The decrease of within-DMN integration was further demonstrated in adult ADHD patients using a network homogeneity method (56) and independent component analysis (57). In addition to within-DMN integration, several studies have shown that ADHD is associated with decreased functional connectivity of the DMN with other non-DMN regions (24, 25). For instance, the dorsal anterior cingulate cortex, a key node of the CON, has consistently been reported to show a decreased negative relationship with the DMN regions in both children (23) and adults (22) with ADHD. These findings are consistent with a recent study demonstrating both short-range and long-range functional connectivity decreases of the DMN in ADHD patients (58). Notably, increased DMN connectivity has also been reported in ADHD (59, 60). Different locations of seeds or clinical features (e.g., drugs) may at least partially account for these discrepancies.

The FPN, also known as the executive control circuit, is involved in sustained attention, inhibition, working memory and goal-directed decision making. There is a wealth of neuroimaging evidence for ADHD-related abnormalities in the structure (e.g., atrophy) and function (e.g., hypo-activation) of the FPN (13, 15, 61, 62). For example, a meta-analysis of 55 fMRI studies revealed convergent findings of hypo-activation of the FPN in ADHD (63). In addition to these local features, abnormal functional connectivity of the FPN has been increasingly reported in ADHD (60, 64, 65). For instance, a very recent study demonstrated that ADHD children had weaker connectivity between the anterior prefrontal cortex and the ventrolateral prefrontal cortex and inferior parietal lobule (65). Consistent with these previous studies, we also observed decreased functional connectivity of the FPN in ADHD. The FPN-related connectivity decreases may account for executive dysfunction such as response inhibition and attentional control in this disease (65). In addition, the CON and CN also exhibited hypoconnectivity in the ADHD children. Taken together, the widespread functional connectivity decreases observed in the present study support the viewpoint of ADHD as a disconnection disease (12).

The most intriguing finding for the current study is that the reduced functional connectivity in ADHD was primarily long-range connections. Long-range connections play vital roles in guaranteeing global integration and generating cognitive function. A previous study showed that, across development, functional connectivity among regions of the FPN, CON, and CN increases for long-range connections but decreases for short-range connections (19). These development-related connectivity changes are further demonstrated among the four networks studied here (26), indicating a general developmental principle for changes in functional connectivity. Accordingly, the decreases of long-range connections observed here provide strong empirical evidence for the standpoint that ADHD is associated with delayed/disrupted functional maturation during the developmental (55, 66). Consistent with the finding of decreased functional connectivity, topological analysis also revealed a less integrative network organization in ADHD, further supporting the delayed/disrupted functional maturation from a local or segregated to distributed or integrated network organization in ADHD. However, after controlling for the lower level of functional connectivity, an increased global efficiency was observed in the ADHD children, implying a more efficient global integration in the patients’ brains at fixed wiring costs. Although the underlying mechanism is unclear, the higher cost-performance may reflect compensatory adaptations of the brain in response to pathological processes induced by ADHD. Notably, for each thresholding approach, the findings were robust when different thresholds were used. The thresholding approach-dependent but thresholding value insensitive findings suggest that absolute and relative network organizations are differentially affected in children with ADHD. This is in line with the notion that different thresholding approaches permit the quantification of distinct aspects of topological network organization.

We found that the decreased functional connectivity in ADHD children exhibited significantly positive correlations with the hyperactivity index and impulsive scores of the patients. This implies that the larger the impulsive and hyperactive scores are for the ADHD children, the stronger the functional interactions are for the children’s control-related brain networks. The counterintuitive brain–behavioral relationships, indeed, have been previously reported in ADHD (67) and other diseases such as schizophrenia (68). Given the highly plastic nature of the human brain in particular during childhood, one possible interpretation is that it is related to compensatory mechanisms of the ADHD children’s brains in response to atypical conditions by adaptively adjusting relevant connectivity strengths and even connectivity patterns. Alternatively, the relationships might also arise incidentally from a common etiologic mechanism, a possibility that has received little attention (69). Notably, the phenomenon of counterintuitive brain–behavioral relationship is poorly understood in brain disorders currently, thus any interpretation should be regarded with caution. Moreover, our ROC analysis showed that the aberrant functional connectivity differentiated the ADHD children from the HCs with high sensitivity and specificity, indicating the potential of imaging-based network analysis in facilitating ADHD diagnosis. In the future, the discriminant ability could be further improved by employing more robust machine-learning methods (e.g., supporting vector machine) or combining multimodal MRI features (70) as well as biochemical and neuropsychological measures in conjunction with clinical variables.

Several issues should be further addressed. First, the cross-sectional design of the current study limited our ability to examine the distinct developmental trajectories of these control-related networks in ADHD children. Future follow-up longitudinal studies will aid in addressing this interesting issue. In particular, studies covering the whole life span of the same patients will be extremely informative to understand how the observed alterations emerge, develop and reorganize at different stages of life and under different conditions in ADHD. Second, ADHD patients can be divided into different subtypes with unique structural and functional connectivity patterns in the brain (71–73). A more fine-grained analysis of the similarities and differences in network topology among these subtypes remains to be performed. Third, previous studies have shown that functional connectivity patterns are largely shaped by structural pathways (74), thus it would be interesting to examine whether the observed functional alterations have underlying structural substrate. Fourth, we demonstrated a less integrative organization of four control-related networks in children with ADHD. Future studies are needed to systematically examine the neural correlates of these alterations with clinical and cognitive characteristics in ADHD. Finally, although the precise etiology of ADHD is unclear, genetic factors have received an increasing amount of attention (75–77). Thus, an important future direction is to establish how ADHD risk genes (e.g., DRD4 and DAT1) modulate brain networks.

## Conclusion

By combining R-fMRI and network approaches, the current study demonstrated a less-integrated architecture of control-related functional brain networks in children with ADHD, which may underlie executive dysfunction of the disease.

## Ethics Statement

This study was approved by the local ethical committee of the First Hospital Affiliated to Wenzhou Medical University. After a complete explanation of the study protocol, all children agreed to take part in this study, and written informed consent was obtained from their guardians. This study was approved by the local ethical committee of the First Hospital Affiliated to Wenzhou Medical University. Written informed consent was obtained from children’s guardians.

## Author Contributions

Designed the experiments: JW, CY, JL, and MW. Performed the experiments: XW, HL, AQ, QY, JT, and HC. Analyzed the data: JT and XJ. Contributed analysis tools: JW. Wrote the paper: JT, JW, and MW.

## Conflict of Interest Statement

The authors declare that the research was conducted in the absence of any commercial or financial relationships that could be construed as a potential conflict of interest.

## Footnotes

^1^http://www.fil.ion.ucl.ac.uk/spm/.

^2^https://irc.cchmc.org/software/tom.php.

^3^http://www.indiana.edu/~cortex/connectivity_toolbox.html.

^4^http://www.mathworks.com/matlabcentral/fileexchange/19950-rocout=roc-varargin-.
